# 20.3 OPEN CHROMATIN ANALYSES INFORM FUNCTIONAL NONCODING GWAS VARIANTS IN HIPSC MODEL OF MENTAL DISORDERS

**DOI:** 10.1093/schbul/sby014.082

**Published:** 2018-04-01

**Authors:** Jubao Duan

**Affiliations:** NorthShore University Health System/University of Chicago

## Abstract

**Background:**

Neuropsychiatric disorders, including schizophrenia (SZ), afflict a significant fraction of the population. Recent genome-wide association studies (GWAS) under the framework of the Psychiatric Genomics Consortium (PGC), along with large-scale sequencing efforts, have identified a plethora of disease risk loci with common and/or rare risk variants. Translating these exciting genomic findings into causation and disease biology offers the promise of developing more tailored therapies in psychiatry. However, understanding the disease biology underlying most GWAS findings remains challenging: (1) The paucity of disease-relevant biological materials for assaying molecular and cellular phenotypes associated with risk loci; (2) Most disease variants lie within poorly-annotated noncoding parts of the genome for which functional interpretation is challenging; and (3) Each locus often contains many genes/variants equivalently associated with the disease due to linkage disequilibrium, and it is difficult to identify which are the likely causal gene/variant. Human neurons derived from induced pluripotent stem cells (iPSCs), both monolayer cultures (2D model) and the emerging brain organoids (3D model), provide a promising alternative to human brains for recapitulating cellular phenotypes relevant to psychiatric disorders. CRISPR/Cas9 editing further strengthens the utility of these models by enabling the generation of isogenic lines with essentially the same genetic background on which allelic effects of a risk variant can be directly compared, thus increasing the sensitivity to detect typically small effects of a GWAS variant.

**Methods:**

To functionally assess the relevance of noncoding sequences in neuropsychiatric disorders, we hypothesized that disease-relevant noncoding sequences likely overlap with cell-specific open chromatin regions (OCRs). We have carried out a genome-wide OCR profiling of excitatory neuronal differentiation from human iPSCs using an Assay for Transposase-Accessible Chromatin by sequencing (ATAC-seq).

**Results:**

We found that OCRs in neurons were enriched SZ risk variants in neural OCRs and can help prioritize putatively functional SZ risk variants that may impact OCRs and consequently, cellular development. At a leading SZ-risk locus flanking MIR137, we further examined the functional effects of a prioritized common GWAS SNP rs1198588 in CRISPR/Cas9-edited hiPSCs, and found that SZ-risk allele of rs1198588 altered MIR137 expression, OCR dynamics and dendrite arborization/synapse maturation. To systematically identify such disease risk variants that may affect OCR, we further carried out a proof-of-concept analysis of allele-specific open chromatin (ASoC) of in hIPSC-derived neurons. We found that Heterozygous SNPs showing ASoC are more prevalent in neurons than in hiPSCs. Out of the 12 schizophrenia GWAS-implicated SNPs that we found in neuronal OCRs of this single individual, two SNPs showed ASoC and are thus putatively functional: one lies within the 5’-UTR of CHRNA5 (cholinergic receptor, nicotinic, alpha 5) and the other is in the promoter region of VPS45, a Sec1 family gene involved in synaptic transmission. We are currently in the process of replicating the observed landscape of ASoC in iPSC-derived neurons from a larger sample pool.

**Discussion:**

Our study suggests that OCR profiling in a human iPSC model of neuron differentiation can predict functional noncoding sequences that regulate neurodevelopment.

